# A phase I/II study of gemcitabine during radiotherapy in children with newly diagnosed diffuse intrinsic pontine glioma

**DOI:** 10.1007/s11060-017-2575-9

**Published:** 2017-07-26

**Authors:** Sophie E. M. Veldhuijzen van Zanten, Fatma E. El-Khouly, Marc H. A. Jansen, Dewi P. Bakker, Esther Sanchez Aliaga, Cornelis J. A. Haasbeek, Nicole I. Wolf, C. Michel Zwaan, W. Peter Vandertop, Dannis G. van Vuurden, Gertjan J. L. Kaspers

**Affiliations:** 1 Department of Pediatric Oncology - Hematology, VU University Medical Center, Amsterdam, The Netherlands; 2 Department of Clinical Pharmacology & Pharmacy, VU University Medical Center, Amsterdam, The Netherlands; 3 Department of Child Neurology, VU University Medical Center, Amsterdam, The Netherlands; 4 Department of Radiology & Nuclear Medicine, VU University Medical Center, Amsterdam, The Netherlands; 5 Department of Radiotherapy, VU University Medical Center, Amsterdam, The Netherlands; 6 Neuroscience Amsterdam, Amsterdam, The Netherlands; 7 Department of Pediatric Oncology - Hematology, Erasmus MC-Sophia Children’s Hospital, Rotterdam, The Netherlands; 8 Neurosurgical Center Amsterdam, VU University Medical Center, Amsterdam, The Netherlands; 9 Neurosurgical Center Amsterdam, Academic Medical Center, Amsterdam, The Netherlands; 10 Princess Máxima Center for Pediatric Oncology, Utrecht, The Netherlands; 11 Department of Pediatrics, Division of Oncology - Hematology, VU University Medical Center, De Boelelaan 1117, Room 9D36, 1081 HV Amsterdam, The Netherlands

**Keywords:** Diffuse intrinsic pontine glioma (DIPG), Radiotherapy, Radiosensitizer, Gemcitabine

## Abstract

The purpose of this phase I/II, open-label, single-arm trial is to investigate the safety, tolerability, maximum tolerated dose and preliminary efficacy of the potential radiosensitizer gemcitabine, administered concomitantly to radiotherapy, in children with newly diagnosed diffuse intrinsic pontine glioma (DIPG). Six doses of weekly gemcitabine were administered intravenously, concomitantly to 6 weeks of hyperfractionated radiotherapy. Successive cohorts received increasing doses of 140, 175 and 200 mg/m^2^ gemcitabine, respectively, following a 3 + 3 dose-escalation schedule without expansion cohort. Dose-limiting toxicities (DLT) were monitored during treatment period. Clinical response was assessed using predefined case report forms and radiological response was assessed using the modified RANO criteria. Quality of life (QoL) was assessed using PedsQL questionnaires. Between June 2012 and December 2016, nine patients were enrolled. Treatment was well tolerated, and no DLTs were observed up to the maximum dose of 200 mg/m^2^. All patients experienced reduction of tumor-related symptoms. QoL tended to improve during treatment. PFS and MOS were 4.8 months (95% CI 4.0–5.7) and 8.7 months (95% CI 7.0–10.4). Classifying patients according to the recently developed DIPG survival prediction model, intermediate risk patients (n = 4), showed a PFS and MOS of 6.4 and 12.4 months, respectively, versus a PFS and MOS of 4.5 and 8.1 months, respectively, in high risk patient (n = 5). Gemcitabine up to 200 mg/m^2^/once weekly, added to radiotherapy, is safe and well tolerated in children with newly diagnosed DIPG. PFS and MOS were not significantly different from literature.

**Electronic supplementary material:**

The online version of this article (doi:10.1007/s11060-017-2575-9) contains supplementary material, which is available to authorized users.

## Introduction

Patients with diffuse intrinsic pontine glioma (DIPG) face a dismal prognosis, with less than 10% of the patients being alive at 2 years after initial diagnosis, and a median overall survival (MOS) of 9 months [1, 2]. To date, radiotherapy is the only effective, albeit palliative, treatment option, with temporary improvement of symptoms and a survival benefit of approximately 3 months [3].

Gemcitabine, a pyrimidine antimetabolite of cytosine, has proven to penetrate the blood–brain–barrier (BBB) reaching radiosensitizing levels in adults with glioblastoma multiforme (GBM), when administered in single doses of 500 or 1000 mg/m^2^ [4]. Furthermore, gemcitabine displays radiosensitizing effects at concentrations 1000 times lower than cytotoxic plasma levels [5]. The radiosensitizing doses in the current trial were based on studies by Fabi et al. [6] and Metro et al. [7], showing a maximum tolerated dose (MTD) of 175 mg/m^2^ gemcitabine once weekly, concomitant to radiotherapy in adult GBM [6, 7]. In children, gemcitabine monotherapy has proven to be safe and tolerable up to cytotoxic dosages of 3600 mg/m^2^/week in leukemia, Hodgkin’s lymphoma and solid tumor patients [8–11]. Toxicities observed in these pediatric studies are mainly myelotoxicity, elevation of liver enzymes and mucositis. Since the present study aims for the radiosensitizing effect of gemcitabine, much lower doses were used, and only limited additional toxicity was not expected.

The purpose of this study is to (i) determine the safety and tolerability of adding the radiosensitizer gemcitabine, to standard radiotherapy in patients with newly diagnosed DIPG, using three pre-specified dose levels, (ii) explore the preliminary efficacy in terms of clinical and radiological response and to compare progression free survival (PFS) and MOS at these dose levels, and to (iii) evaluate the quality of life (QoL) during treatment.

## Patients and methods

### Approval

This study (EudraCT 2009-016080-11, Dutch Trial Register NTR2391) was approved by the institutional review board of VU University Medical Center (METc VUmc, study number: VUMC2010/164), and the Scientific Committee of the Dutch Childhood Oncology Group (DCOG). The use of gemcitabine has been approved by U.S. Food and Drug Administration (FDA) and European Medicines Agency (EMA) for adults, and has been tested and proven to be safe in children up to 1000 mg/m^2^/dose (off-label) when combined with other chemotherapeutic drugs [12]. All parents signed informed consent, and patients between 12 and 18 years of age also signed informed assent.

### In- and exclusion criteria

Children aged 3–18 years with newly diagnosed DIPG were eligible for this study. Inclusion criteria were: (i) diagnosis of a typical DIPG: symptoms <6 months and MRI-confirmed (≥50% involvement of the transverse area of the pons, T1 hypointensity, T2 hyperintensity, with a clear origin in the pons), (ii) written informed consent, (iii) transfusion-independent platelet count ≥75 × 10^9^/L, peripheral absolute neutrophil count (ANC) ≥0.75 × 10^9^/L, (iv) adequate liver function, defined as direct bilirubin ≤1.5 × upper limit of normal (ULN) for age and alanine aminotransferase (ALAT) <5 × upper limit of normal (ULN) for age, (v) adequate renal function, defined as serum creatinine ≤1.5 × upper limit of normal (ULN) for age, (vi) willingness to perform a pregnancy test and apply contraceptives in females of child-bearing age. Biopsy was offered as an option, but was not mandatory for the diagnosis of a DIPG. Exclusion criteria were: (i) clinically-diagnosed neurofibromatosis (NF) type I (DNA-diagnostics not mandatory), (ii) patients who received prior therapeutic treatment for DIPG (except corticosteroids), (iii) presence of diffuse leptomeningeal disease, (iv) performance status (Lansky or Karnofsky score) of 40 or less (v) contra-indications for chemotherapy.

### Study objectives

The primary objective was to determine the safety and tolerability of gemcitabine at three pre-specified different dose levels; 140, 175 and 200 mg/m^2^. The secondary objective was to evaluate the preliminary efficacy in terms of PFS and MOS at these dose levels. Progressive disease was defined as clinical signs of disease progression (i.e., increase of symptoms or new symptoms) and/or radiological progression based on the modified RANO criteria. The tertiary objective was to evaluate the quality of life (QoL) using QoL questionnaires.

### Study procedures

Patients received weekly gemcitabine, concomitant with 6 weeks of radiotherapy: 54 Gy in 30 fractions of 1.8 Gy directed at the tumor using volumetric modulated arc therapy (VMAT). The VMAT technique was used to reduce toxicity as much as possible. Gemcitabine was administered intravenously once weekly for 6 weeks, starting 24 h before the first day of radiotherapy. This schedule was based on a previous gemcitabine and radiotherapy study [6, 7]. Doses were escalated in successive patients following a traditional 3 + 3 dose-escalation, without a dose expansion cohort [13]. If a dose limiting toxicity (DLT) was observed in one out of three patients in a specific cohort, three additional patients were enrolled in that cohort. The MTD was reached if more than one out of six patients (in 1 cohort) developed a DLT. In that case further dose escalation was not pursued. If no DLT was observed in any of the patients in a specific cohort at 2 weeks after gemcitabine administration, additional trial patients were treated following the next higher dose cohort until the predefined highest dose of 200 mg/m^2^ was reached [13]. Following this method, a minimum of three and a maximum of 18 patients were expected to be included. The starting dose was 140 mg/m^2^, which is 80% of the recommended dose of 175 mg/m^2^ by Fabi et al. [6, 7]. Successive cohorts received doses of 175, and 200 mg/m^2^ as the predefined highest dose. Gemcitabine was reconstituted in 50 mL 0.9% sodium chloride solution and administered intravenously at a rate of 10 mg/m^2^/min. Prior to each cycle of gemcitabine, patients were required to qualify based on hematological examination; the ANC had to be equal to or >0.75 × 10^9^/L, and the platelet count equal to or >75 × 10^9^/L. If required, ondansetron (Zofran^®^) was administered intravenously before gemcitabine administration or orally before radiotherapy. All patients were treated in one center, VU University Medical Center (VUMC) Amsterdam.

#### Safety assessments and response evaluation

A DLT was defined as any clinically relevant, and likely drug-related, grade ≥3 adverse event (AE), according to criteria outlined in the NCI Common Terminology Criteria for Adverse Events (CTCAE), version 4.0 [14]. Asymptomatic laboratory abnormalities were not considered a DLT. DLTs were evaluated during the 6 weeks treatment period.

Safety assessments (i.e., evaluation of DLTs) included weekly examination of urine and blood (hemoglobin), platelets, white blood cell count and differentiation, creatinine, blood urea nitrogen, uric acid, albumin, sodium, potassium, calcium, magnesium, phosphate, ASAT, ALAT, G-GT, bilirubin, LDH, bicarbonate, glucose, and extra collections according to the treating physician. Urine and blood were examined weekly during the 6-week treatment period. In case of abnormalities, safety assessments proceeded until aberrant values normalized.

Additionally, patients underwent regular full physical and neurological examination by either a pediatric oncologist or a child neurologist in order to assess possible DLTs and possible disease progression. This was performed once every 2 weeks during treatment to assess DLTs, and for disease progression once monthly until 3 months post-treatment (i.e. week 19) or until disease progression. In case of stable disease after week 19, clinical follow-up was performed every 3 months to determine the PFS and MOS. Disease progression was defined as clinical signs of disease progression (i.e., increase of symptoms or new symptoms) and/or radiological tumor progression, whichever came first.

Radiologic response was evaluated by a neuro-radiologist using the modified RANO criteria, which takes into account the change in T1 gadolinium enhancement and T2 tumor size, development of metastasis, use of corticosteroids and clinical status [15]. MR-scans of the brain and spinal cord were performed at baseline, 3 months post-treatment and/or at disease progression.

QoL was assessed at baseline, and 3 months post-treatment by three categories of the PedsQL questionnaires (Pediatric Quality of Life Inventory TM [16, 17]): (i) the PedsQL TM 4.0 Generic Core Scales, addressing physical performance and psychosocial health, (ii) the PedsQL TM Multidimensional Fatigue Scale, addressing general fatigue, sleep rhythm and cognitive fatigue and (iii) the PedsQL TM 3.0 Cancer Module, addressing pain during treatment, nausea, fear of treatment and procedures, worrying about disease course and appearances and communication with other people. PedsQL provides age-appropriate questionnaires that take approximately 10 min per category.

### Supportive care

In the first week of radiotherapy, the use of dexamethasone was allowed in order to reduce symptoms related to edema formation. After the first week, physicians were encouraged to stop or taper dexamethasone as soon as possible because of associated side-effects [18] and possible negative effects on blood–brain–barrier penetrance and activity of gemcitabine [19, 20].

### Statistical methods

Data were analyzed using IBM SPSS Statistics for Windows, Version 22.0 (Armonk, NY: IBM Corp. Released 2013). Patient data regarding demographics were analyzed by descriptive statistics. PFS and MOS were determined by means of Kaplan–Meier method. PFS and MOS of the total study cohort were compared to historical survival data of DIPG patients receiving “radiotherapy only” found in literature. Subsequently, each patient was classified in a risk-category according to the recently developed DIPG survival prediction model, to evaluate whether the risk-classification of the patients might have influenced the observed survival [21, 22].

For each patient, an individual risk-score was calculated based on the following formula: symptom duration (in months) at time of diagnosis (time minus one), added with seven points if age ≥3 years, added with four points if ring enhancement is present on diagnostic MRI. Based on the risk-score, patients were categorized as being standard- (score ≤1), intermediate- (1–6) or high-risk (≥7). For each risk-group subgroup specific PFS and MOS were calculated, and compared to the survival data reported by Jansen et al. [21] QoL data before and after treatment were compared using the Wilcoxon signed ranks test. Statistical significance was defined as a 2-sided *P* < 0.05.

## Results

### Patients

Between June 2012 and December 2015, nine patients with newly diagnosed DIPG were included in this study. Patient data regarding demographics, clinical characteristics and diagnosis are summarized in Table 1. The median age was 10.8 years (range 7.5–17.3). Signs of disease preceded the hospital presentation by a median time of 2 weeks. Symptoms most commonly observed were abducens nerve palsy, diplopia, impaired coordination and ataxia. None of the patients received any treatment prior to participating in this study. Based on the DIPG survival prediction model, four patients were intermediate- and five patients were high-risk patients, with scores varying from 6.0 to 10.75 [21].

**Table 1:** Baseline characteristics

Patient ID	Age at diagnosis (years)	Gender	Histology	Encasement a. Basilaris	Ring enhancement	Metastases	Symptom duration (weeks)	Dexa use	Risk group	Study cohort
1	17.3	F	Anaplastic Astrocytoma (WHO III)	181 < encasement < 360°	Yes	No	4	No	High	1
2	11.8	M	n.a.	181 < encasement < 360°	Yes	No	2	Yes	High	1
3	11.2	M	Glioblastoma (WHO IV)	181 < encasement < 360°	Yes	No	1.5	Yes	High	1
4	10.8	M	n.a.^a^	181 < encasement < 360°	No	No	4	Single dose	Intermediate	2
5	12.4	F	Glioblastoma (WHO IV)	Full (360°)	No	No	0.5	Yes	Intermediate	2
6	7.5	M	Astrocytoma (WHO II)	181 < encasement < 360°	No	No	2	No	Intermediate	2
7	7.6	F	n.a.	181 < encasement < 360°	Yes	No	3	No	High	3
8	7.7	M	n.a.	Full (360°)	No	No	2	No	Intermediate	3
9	9.9	F	n.a.	Full (360°)	Yes	No	1	Yes	High	3
Median	10.8						2.0			

*n.a*. not applicable, meaning no biopsy performed

^a^Biopsy performed, inconclusive results

### Toxicity

All patients received radiotherapy up to a total dose of 54 Gy following the predefined schedule of 30 fractions of 1.8 Gy. Cohort 1, 2 and 3, received gemcitabine once weekly for 6 weeks, in doses of 140, 175 and 200 mg/m^2^, respectively. No DLTs, SUSARs or SAEs occurred.

In each cohort, the minimum of three patients was included since radiotherapy and concomitant gemcitabine were well tolerated. No grade 4 or 5 laboratory toxicities were reported. Two patients showed laboratory grade 3 hepatotoxicity: an increase in ALAT during their 6 weeks of treatment, up to 241 U/L in week 5, and 227 U/L in week 6, respectively (normative value 0–35 U/L). Laboratory grade 2 and 3 neutropenia was observed in two patients. None of the aberrant values had consequences for the clinical well-being of the patient, and all values normalized directly after treatment without any intervention. Therefore, no clinically relevant grade 3 toxicities were reported.

All patients experienced grade 2 nausea and vomiting during the 6-week treatment period. Therefore, ondansetron (Zofran^®^) was administered intravenously before gemcitabine administration, and additional oral ondansetron was administered if needed before radiotherapy. Nausea and vomiting disappeared directly after treatment was completed. Local alopecia, restricted to the target area of the radiation beam, was observed in all patients. Five out of nine patients experienced enhanced smell and altered taste during treatment, rated as grade 1 toxicity.

### Efficacy

At diagnosis, clinical symptoms associated with DIPG such as ataxia, walking disturbances, and abducens nerve palsy resulting in diplopia were observed in most patients. Four out of nine patients received dexamethasone directly at time of diagnosis. Upon treatment, dexamethasone was tapered in three, and continued in one patient at a low dose (0.25 mg/day coming from 2 × 2 mg/day). One patient received a single dose of dexamethasone during treatment. In all patients, the clinical symptoms improved during treatment. No difference in clinical response was observed between the dose-level cohorts.

In the radiological response evaluation by the neuro-radiologist at 3 months post-treatment, patients four and six had stable disease (SD), based on the modified RANO response criteria [15]. Two patients (patient three and nine) had progressive disease (PD) based on the occurrence of metastases. In the first, metastases spread diffusely via the leptomeninges, infra- and supratentorial, along the spinal cord and intraventricular. In the second, leptomeningeal spread was observed. Four patients showed a pattern fitting either progressive disease or pseudo-progression (PD/psPD). Upon clinical/radiological follow-up, these patients (1, 2, 7, and 8) were retrospectively classified as having PD based on clinical and/or further radiological progression. Finally, one patient showed PD within a month after finishing treatment with the occurrence of metastases (the MRI showed a metastasis in the septum pellucidum and diffuse leptomeningeal spread), and died 1.5 months after treatment (no week 19 scan was made). Supplementary Table 1 provides radiologic response assessments at baseline and 3 months post-treatment. No difference in radiological response was observed between the dose-level cohorts.

**Fig. 1:**
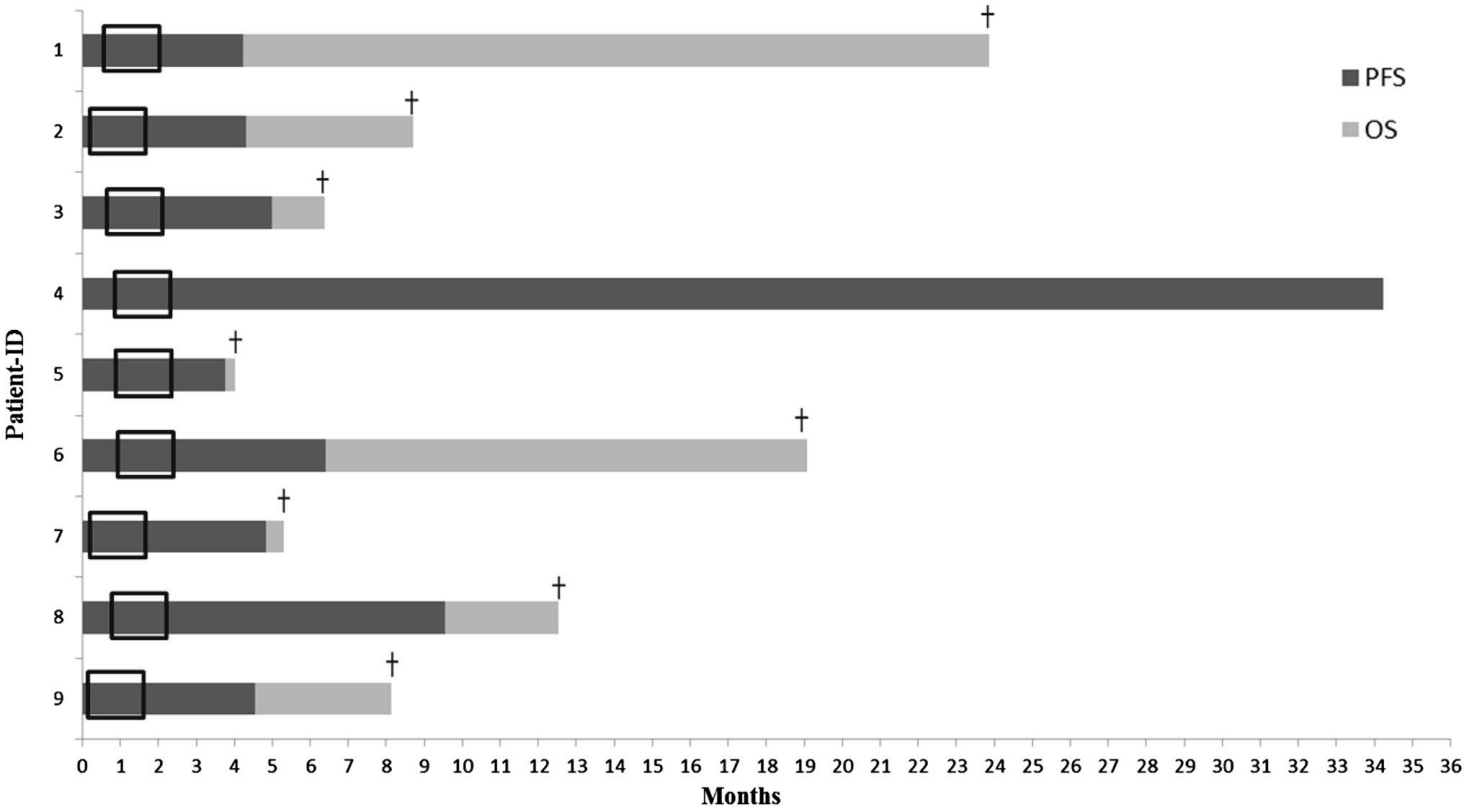
Detailed disease course for each patient included in this study. The frame marks the treatment period of 6 weeks

The median overall PFS and MOS of all nine patients were 4.8 months (95% CI 4.0–5.7) and 8.7 months (95% CI 7.0–10.4), respectively (Supplementary Fig. 1). According to the DIPG survival prediction model, our cohort included four intermediate- and five high-risk patients. PFS and MOS for intermediate-risk patients were 6.4 and 12.4 months, respectively, and for the high-risk patients 4.5 and 8.1 months, respectively (Supplementary Fig. 1). Figure 1 shows a detailed course of the disease for each patient. The median time from diagnosis to start of treatment was 18 days (range 8–28). One patient has not experienced disease progression and is alive at 34.2 months post-diagnosis (March 2017). No difference in survival was observed between the dose-level cohorts.

### Quality of Life

Table 2 contains total scores per questionnaire-category, and separate scores per subcategory within the questionnaire-categories. The higher the score, the better the QoL. All patients scored relatively high on all categories of the PedsQL TM 4.0 Generic Core Scales and the PedsQL TM Multidimensional Fatigue Scale questionnaires, except on the psychosocial health, which includes school absence. Although not statistically significant, all median scores of the PedsQL TM 4.0 Generic Core Scales questionnaire increased, suggesting a better quality of life after treatment. Furthermore, the PedsQL TM Multidimensional Fatigue Scale scores decreased after treatment. In the subcategories of the PedsQL TM 3.0 Cancer Module questionnaire, nausea and fear of procedure scored significantly lower after treatment compared to baseline. This resulted in a significant decrease in the total score of the cancer questionnaire (*P* = 0.042). No difference in quality of life was observed between the dose-level cohorts.

**Table 2:** Quality of Life assessment

	Median [IQR]		*P* value (<0.05)
	Week 0	Week 19	
PedsQL TM 4.0 Generic Core Scales			
Self-report			
Physical performance	87.50 [37.50;96.88]	85.94 [64.06;93.75]	0.854
Psychosocial health	85.00 [73.33;86.67]	87.50 [76.67;93.33]	0.273
Total score	82.61 [60.87;86.96]	84.78 [74.46;93.48]	0.715
Parent report			
Physical performance	78.13 [28.13;90.63]	82.81 [62.50;86.72]	0.715
Psychosocial health	44.57 [42.39;54.35]	48.91 [46.47;49.73]	0.144
Total score	44.57 [42.39;54.35]	48.91 [46.47;49.73]	0.144
PedsQL TM Multidimensional Fatigue Scale			
Self-report			
General score	75.00 [58.33;89.58]	83.33 [75.00;91.67]	0.713
Sleep score	79.17 [56.25;97.92]	83.33 [70.83;93.75]	0.414
Cognitive fatigue score	79.17 [66.67;100.00]	83.33 [62.50;97.92]	0.713
Total score	81.94 [61.81;88.19]	81.94 [73.61;90.97]	0.500
Parent report			
General score	66.67 [50.00;77.08]	87.50 [68.75;93.75]	0.273
Sleep score	83.33 [54.17;93.75]	83.33 [83.33;95.83]	0.461
Cognitive fatigue score	75.00 [56.25;89.58]	95.83 [77.08;97.92]	0.141
Total score	81.94 [54.86;84.72]	86.11 [81.11;92.36]	0.080
PedsQL TM 3.0 Cancer Module			
Self-report			
Pain	100.00 [100.00;100.00]	100.00 [75.00;100.00]	0.157
Nausea	100.00 [100.00;100.00]	80.00 [60.00;90.00]	0.042
Fear of procedures	100.00 [100.00;100.00]	16.67 [4.17;50.00]	0.042
Fear of treatment	100.00 [100.00;100.00]	75.00 [54.17;100.00]	0.109
Worry	100.00 [100.00;100.00]	83.33 [75.00;100.00]	0.102
Cognitive functioning	100.00 [100.00;100.00]	75.00 [50.00;100.00]	0.180
Appearance	100.00 [100.00;100.00]	100.00 [87.50;100.00]	0.180
Communication	100.00 [100.00;100.00]	83.33 [70.83;100.00]	0.180
Total score	100.00 [100.00;100.00]	77.78 [69.44;80.56]	0.042
Parent report			
Pain	100.00 [100.00;100.00]	100.00 [87.50;100.00]	0.317
Nausea	95.00 [90.00;100.00]	65.00 [40.00;85.00]	0.104
Fear of procedures	112.50 [75.00;150.00]	25.00 [00.00;87.50]	0.041
Fear of treatment	100.00 [70.83;100.00]	75.00 [54.17;100.00]	0.109
Worry	100.00 [87.50;100.00]	75.00 [75.00;100.00]	0.157
Cognitive functioning	80.00 [77.50.00;84.38]	60.00 [55.63;75.00]	0.068
Appearance	100.00 [87.50.00;100.00]	83.33 [75.00;95.83]	0.066
Communication	100.00 [83.33.00;100.00]	100.00 [87.50;100.00]	0.18
Total score	94.23 [86.57;96.71]	72.22 [68.32;81.02]	0.043

## Discussion

This phase I/II open-label single arm trial demonstrates that conventionally-fractionated radiotherapy (54 Gy), combined with weekly gemcitabine in a dose up to 200 mg/m^2^/week, is safe and well tolerated in children aged 7–17 years with newly-diagnosed DIPG. PFS and MOS were not different from historical control patients from equal risk-score cohorts. QoL tended to improve, but not to a statistically significant extent.

In previous studies in children, in which up to 18-fold higher doses of gemcitabine were given without radiotherapy, myelotoxicity was reported, as well as elevation of liver enzymes and mucositis [8–11]. In our study, no DLTs, defined as clinically relevant grade 3 toxicities, were observed. During the 6-week treatment period, only decreased neutrophil counts and elevated ALAT were observed. These aberrant laboratory values all normalized directly after treatment and did not require treatment interruption or other medical management. By using the VMAT technique, toxicity of the irradiated mucosa was prevented and no mucositis was observed. All patients reported nausea and vomiting, occurring directly after gemcitabine administration and during radiotherapy, which was considered the main disadvantage of participation in this study.

Efficacy, in terms of clinical and radiological response, PFS and MOS, was not different when compared to historical control patients from equal risk-score cohorts. In patients with psPD, the clinical course of the patients provided the distinction between PD and psPD. Possibly, radiologic responses could have been more conclusive if successive MR-scans had been made with shorter time-intervals.

The overall PFS and MOS found in this study, were relatively short (4.8 and 8.7 month, respectively) compared to survival data of DIPG patients receiving “radiotherapy only” found in literature (i.e. PFS of 6.0 months, MOS of 10.0 months) [23]. This could be explained by the fact that all patients included in this study were either intermediate- (four) or high-risk (five) patients, when classified according to the recently developed and validated DIPG survival prediction model [21, 22]. In the first study, intermediate- and high-risk DIPG patients showed a PFS of 7.0 and 5.0 months, respectively (unpublished data), and a MOS of 9.7 and 7.0 months, respectively. It should be taken into account, however, that this cohort included patients that where treated with radiotherapy only, but also patients that received successive treatments after disease progression. Direct comparison of survival times might therefore not be reliable, but the observed difference between the risk-groups is comparable in both studies and could be relevant in the interpretation of our results. Finally, in contrast to the relative short MOS of the total cohort, our study cohort includes two so-called long-term survivors (arbitrarily defined as patients who survive ≥24 months post-diagnosis). Both patients were typical DIPG patients based on their clinical symptoms and MR-imaging characteristics at time of diagnosis. Histology from biopsy of one of these patients showed WHO grade III anaplastic astrocytoma. Results of the other long-term survivor were, unfortunately, inconclusive and thus far repeat biopsy has not been performed. The patient showing WHO grade II astrocytoma histology had a PFS of 6.4 months, in accordance with literature, and a relatively long OS of 19.1 months. Since it is well known that DIPGs are heterogeneous tumors [24], with areas varying from low (WHO grade II) to high-grade (WHO grade III and IV) tumors, we did not take histology into account in the interpretation of our results. Moreover, WHO grade II–IV was previously found not to correlate with survival in DIPG [25]. Determination of histone mutation status would be of interest, but is not known for the patients included in this study, as the analysis was not readily available when the study was designed in 2012. However, the prolonged survival (i.e. >18 months) of three (out of nine) patients might be caused by a different underlying tumor biology. To conclude, with the current study design with limited patient numbers, it cannot be determined whether the prolonged survival of these patients is a result of the treatment itself, or influenced by the underlying biological background of their tumors.

Current knowledge about the BBB in DIPG suggests that gemcitabine has limited BBB passage. However, as radiotherapy has been shown to temporarily disrupt the BBB, administration of gemcitabine is expected to be more efficient and effective during radiotherapy [26]. Based on recent studies, dosages up to 750 mg/m^2^ can safely be explored in future gemcitabine-radiotherapy studies [27]. Potentially gemcitabine dosing in combination with radiotherapy can be escalated even further, as in children dose limiting toxicities of gemcitabine monotherapy were only observed at doses as high as 3600 mg/m^2^. Since these DLTs were mainly hepatotoxicity and hematological toxicity, it is unlikely that local radiotherapy to the brainstem will aggravate or increase the incidence of these DLTs.

In vitro studies have described a decreased activity of gemcitabine when combined with dexamethasone [19, 20]. Unfortunately, due to the limited number of patients enrolled in this study and the different treatment schedules used, it is not possible to determine whether dexamethasone use in the current study affected the outcome. Of specific note, in four out of nine patients, including one of the long-term survivors, no dexamethasone was given at diagnosis or during chemo-radiotherapy, and the other long-term survivor used only a single dose of dexamethasone during treatment. This indicates that corticosteroids may not be a stringent necessity in newly diagnosed DIPG patients.

In the assessment of QoL in DIPG patients under treatment, our study showed remarkably low scores for psychosocial health compared to the other scores assessed in this study. This could be attributed to the fact that one question assesses school absence, and due to the intensive treatment schedule most children did not attend school during the 6-week treatment period. The observed decrease in the PedsQL TM 3.0 Cancer Module questionnaire scores might partly be explained by nausea, being the main side effect of this treatment. Off note, to reduce edema and to control nausea during radiotherapy, dexamethasone is commonly used in DIPG patients. In this study, however, the use of dexamethasone was limited because of the possible drug-interaction with gemcitabine, its negative effects on blood–brain–barrier passage of drugs [19, 20] and its well-known side effects [18]. Instead, nausea was treated with ondansetron (Zofran^®^). The observed mild decrease in the PedsQL TM 3.0 Cancer Module questionnaire scores might furthermore be influenced by anxiety around the weekly placement of the IV cannula for the infusion of gemcitabine. In future trials this could be prevented by placement of a central line. Unfortunately, no historical data on QoL are available for DIPG patients. Nevertheless, all patients and parents evaluated participation in this study as a positive experience. Even though this treatment had a great impact on a family’s daily routine for 6 weeks, none of the parents or patients experienced a significant burden from the procedures. Instead, families reported to have experienced support from the intensive level of support and care from the treating physician and the research team. A limitation of the QoL evaluation in this study is that QoL was only measured at two time points (baseline and 3 months after treatment), while longer follow-up could have provided more information. In the design of the study we took into account the burden these questionnaires might have, the duration of administration of the study drug (i.e. 6 weeks), and the short survival of DIPG patients. Recently, Mandrell et al. were the first to demonstrate that frequent evaluation and follow-up of QoL is feasible in DIPG patients [28]. Unfortunately, in that study other types of PedsQL questionnaires were used. Therefore, no one-on-one comparison could be made with the results found in our current study.

In conclusion, this study demonstrates that weekly gemcitabine concomitant to radiotherapy is safe and well tolerated in doses up to 200 mg/m^2^ in children suffering from newly diagnosed DIPG. Because of the limited number of patients included in this study, and the limited knowledge on the biology of each patient’s tumor, no definite conclusions on the preliminary efficacy can be drawn. Based on promising results from recent studies in adult glioblastoma [27], a study to assess the safety, tolerability and efficacy of higher doses of gemcitabine, in combination with radiotherapy, is being prepared. Furthermore, investigating the safety, tolerability and efficacy of the use of gemcitabine concurrently as a radiosensitizer and adjuvant as a cytotoxic therapy could be of interest.

## Electronic supplementary material

Below is the link to the electronic supplementary material.

**Supplementary Table 1** Radiologic response assessment. *n.a.: not applicable, meaning patient died before week 19*. **Supplementary Figure 1** Kaplan-Meier curves showing (A) PFS and MOS for the total cohort, (B) PFS per risk-group, and (C) MOS per risk-group. (DOCX 157 KB)
