# Supplementary material for: A phase I/II study of gemcitabine during radiotherapy in children with newly diagnosed diffuse intrinsic pontine glioma
Source: J Neurooncol. 2017 Jul 26;135(2):307–15. doi: 10.1007/s11060-017-2575-9 (PMC5663796; doi:10.1007/s11060-017-2575-9)
Supplement: Supplementary file 1 — Supplementary Table 1 Radiologic response assessment. n.a.: not applicable, meaning patient died before week 19. Supplementary Figure 1 Kaplan-Meier curves showing (A) PFS and MOS for the total cohort, (B) PFS per risk-group, and (C) MOS per risk-group. (DOCX 157 KB) [file 11060_2017_2575_MOESM1_ESM.docx]

**Supplementary Table 1:**

**Supplementary Figure 1:**

| **A. Overall PFS and MOS of nine patients** | **B. PFS – risk group specific** | **C. MOS – risk group specific** |
| --- | --- | --- |
| **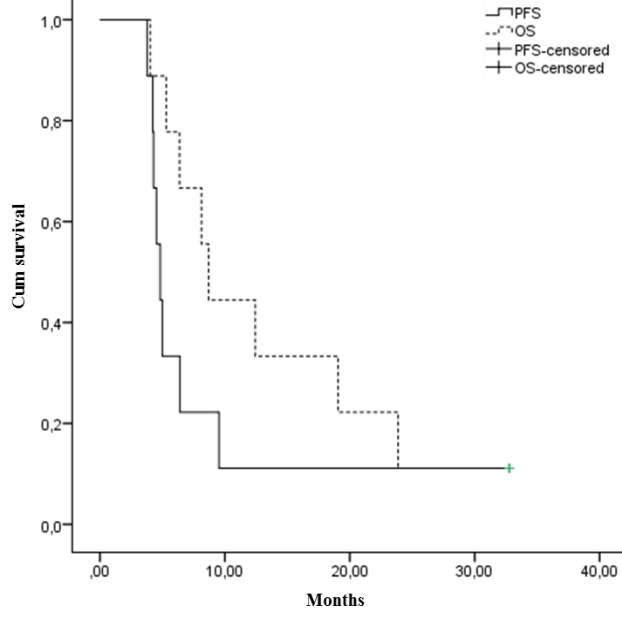** | **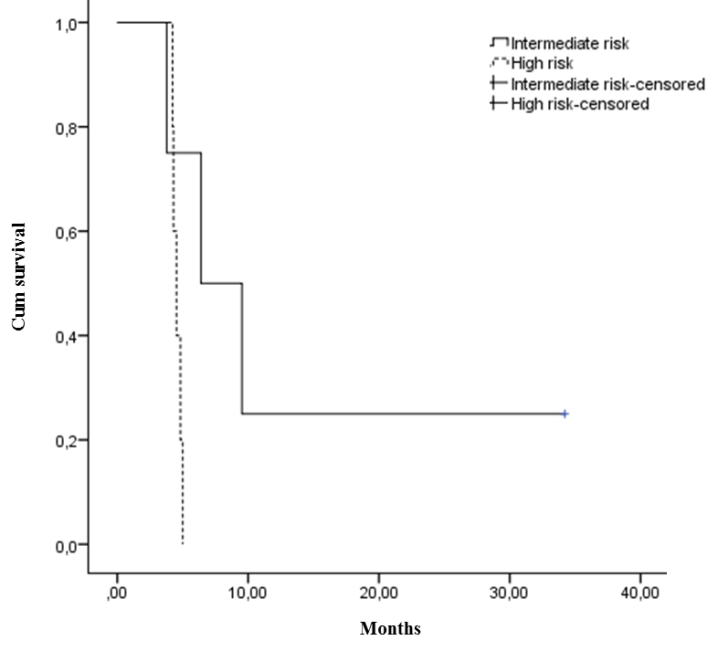** | **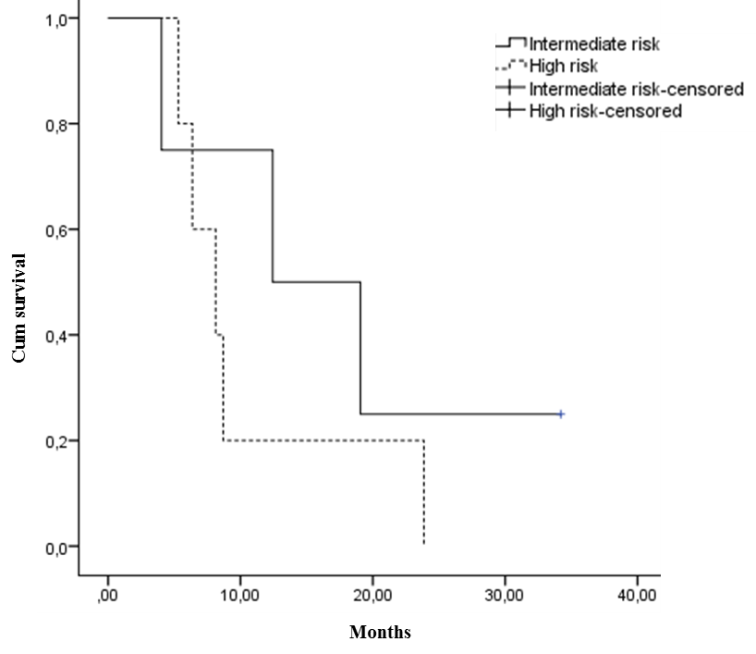** |
